# *Mycobacterium tuberculosis* pseudo-outbreak due to laboratory cross-contamination: A molecular epidemiology outbreak investigation

**DOI:** 10.14745/ccdr.v50i12da03

**Published:** 2024-12-05

**Authors:** Nayla Léveillé, Floriane Point, Josée Houde, Michael Hall, Hafid Souhaline, Marie-Andrée Leblanc, Pierre-Marie Akochy, Simon Grandjean Lapierre

**Affiliations:** 1 Faculté de Médecine, Département de Médicine, Université de Montréal, Montréal, QC; 2 Immunopathology Axis, Centre de recherche du Centre hospitalier de l’Université de Montréal, Montréal, QC; 3 Microbiology Department, Centre hospitalier de l’Université de Montréal, Montréal, QC; 4 European Bioinformatics Institute (EMBL-EBI), Cambridgeshire, United Kingdom; 5 Peter Doherty Institute for Infection and Immunity, University of Melbourne, Melbourne, Australia; 6 National Microbiology Laboratory, Public Health Agency of Canada, Winnipeg, MB; 7 Direction générale adjointe de la protection de la santé publique, Ministère de la Santé et des Services sociaux, Québec, QC; 8 Département de mycobactériologie, Laboratoire de santé publique du Québec (LSPQ), Québec, QC; 9 Département de Microbiologie, infectiologie et immunologie, Faculté de médecine, Université de Montréal, Montréal, QC

**Keywords:** tuberculosis, next-generation sequencing, contamination, outbreak, transplantation

## Abstract

**Background:**

Mycobacterial culture is routinely performed to diagnose tuberculosis (TB) in Canada. Globally, meta-analyses suggest that up to 2% of positive cultures are falsely positive for *Mycobacterium tuberculosis* due to laboratory cross-contamination. Five patients from distinct clinical institutions in Montréal were diagnosed with culture-positive TB as their clinical samples were processed in a centralized mycobacteria laboratory. Cross-contamination was suspected due to culture positivity in an organ donor with low TB pre-test probability. We describe a TB pseudo-outbreak due to laboratory cross-contamination and assess the role of conventional typing (i.e., mycobacterial interspersed repetitive unit variable number of tandem repeats [MIRU-VNTR]) and whole-genome sequencing (WGS) in supporting the investigation.

**Methods:**

Patients’ epidemiological risk factors and clinical presentations were reviewed. The trajectories of pre- and per-analytic samples were retraced to identify potential cross-contamination events. Tuberculosis isolates were characterized by MIRU-VNTR and WGS using Oxford Nanopore Technology (ONT). The bioinformatic pipeline tbpore (v0.7.1) cluster was used for phylogenetic analyses.

**Results:**

Two patients had previous exposure to endemic settings and clinical symptoms compatible with TB. Culture media inoculation overlapped in time for four patients, including one with suspected pulmonary cavitary disease and an organ donor whose organs had been transplanted in three different receivers. The MIRU-VNTR and WGS typing confirmed isolates from those four patients to be identical.

**Conclusion:**

Clinical, laboratory and molecular typing data, including results from ONT sequencing, were considered sufficiently robust to confirm laboratory cross-contamination and TB therapy was discontinued including in all organ transplant recipients.

## Introduction

According to the Public Health Agency of Canada (PHAC), 1,971 cases of active tuberculosis (TB) were reported in 2022, with an associated incidence of 5.1 TB cases per 100,000 people, which has been stable in the last 20 years ((1,2)). In every Canadian province and territory, TB is a mandatory reportable disease and mandatory treatment is enforced by public health authorities in cases of contagious respiratory active disease ((3,4)). Laboratory diagnosis of TB, therefore, has significant clinical and public health implications.

Laboratory cross-contamination was previously reported as a cause of false positive *Mycobacterium tuberculosis* culture. A systematic review published in 2019, including 31 articles regrouping 29,839 TB cultures, suggested that 2% of positive culture results were false positives. For positive cultures from patients with a prior negative sample or negative follow-up sample, the rate of false positives was increased to 15% ((5)). Contamination events are increasingly reported due to wider availability of bacterial genotyping which facilitates the investigation of contamination events ((6)). Similarly for person-to-person transmission and outbreak investigations, restriction fragment length polymorphism (RFLP) and mycobacterial interspersed repetitive unit variable number of tandem repeats (MIRU-VNRT) methods are increasingly replaced by higher resolution whole-genome sequencing (WGS) as the method of choice for molecular genotyping ((6–10)).

We report on a *M. tuberculosis* laboratory contamination event and pseudo-outbreak that has had significant clinical and public health impacts, including among organ transplant patients. We detail our epidemiology and molecular investigation approach, which included patients’ clinical assessments and bacterial DNA sequencing using the Oxford Nanopore Technologies (ONT) next-generation sequencing (NGS) platform and publicly available adapted bioinformatic analysis tool, tbpore cluster (version 0.7.1) ((11)). Ways to further mitigate the risk of future contamination events are also proposed.

## Results

### Setting and participants

The *Centre hospitalier de l’Université de Montréal* (CHUM) is a 700-bed quaternary care hospital providing care to specific populations at higher risk of mycobacterial infections, including cystic fibrosis, lung and other organ transplants, and oncology patients. Our Biosafety Level 3 clinical mycobacteria laboratory processes thousands of samples every year and performs mycobacterial smear microscopy, culture and nucleic acid amplification testing (NAAT) for species of the *M. tuberculosis* complex. All positive cultures are referred to our provincial reference laboratory (*Laboratoire de santé publique du Québec*, LSPQ), where sequencing-based speciation and phenotypic drug susceptibility testing is performed, if indicated.

### Investigation

A false positive *M. tuberculosis* culture result was initially suspected by a clinical infectious disease physician from CHUM in 2023. The positive sample had been collected during lung harvesting from a deceased organ donor. Such cultures are routinely performed to establish pre-transplant donor organ colonization and guide post-transplant receiver empiric antimicrobial therapy. This organ donor had been clinically screened, and in the absence of epidemiological risk factors, was considered to have a null pre-test probability for TB. Tuberculosis infection screening with either tuberculin skin test or interferon-gamma release assay (IGRA) is not routinely performed among organ donors because of low TB incidence in Québec organ donors. The 48-hour minimum assay turnaround time is also frequently too long in the context of urgent post-mortem organ harvesting. This single donor was involved in pulmonary, cardiac, and renal transplantations to three distinct patients receiving post-transplant medical care in three different institutions.

Following clinical suspicion of a false positive result, laboratory, clinical and public health investigations were performed in collaboration with Québec Transplant, the patients’ attending physicians, regional and provincial public health authorities, and the LSPQ reference laboratory. All culture or NAAT-positive samples received, inoculated and processed upon positive culture signal in our laboratory within a three-day timeframe were identified as potential index- or co-contaminated samples. Additionally, patients with positive cultures that were reprocessed with the suspected false positive sample during positive culture media manipulation, and patients with positive cultures that were processed for referral to LSPQ on overlapping periods with the suspected false positive sample, were also identified as potential index- or co-contaminated samples. This approach highlighted specific pre- (prior to sample reception in the laboratory) and per- (during sample processing in the lab) analytical potential cross-contamination events, including those previously described in the literature when various samples are manipulated at the same time (i.e., culture inoculation, positive mycobacteria growth indicator tube [MGIT] media manipulation, aliquoting and shipping) ((5,12)). This allowed us to identify four additional patients as potential index- or co-contaminated samples. Clinical characteristics, epidemiological risk factors and sample trajectories of these five individuals (n=1 organ donor, n=4 contemporary positive samples) were reviewed from clinical and public health charts and laboratory information systems. Twenty-four loci MIRU-VNTR typing was performed by the PHAC National Microbiology Laboratory (NML) and ONT-based WGS was performed in CHUM for each of these samples ((11,13,14)).

### Investigation outcomes

**Figure 1:**
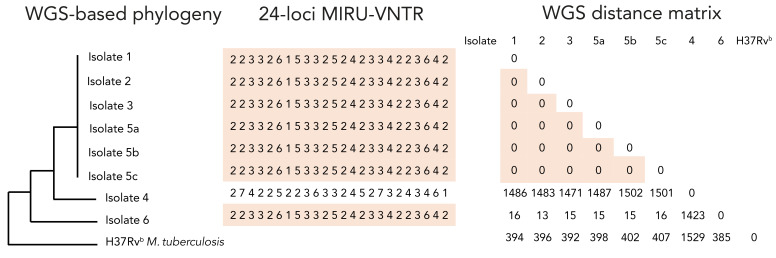
Whole-genome sequencing and MIRU-VNTR typing of putative cross-contamination isolates^a,b^ Abbreviations: *M.*, *Mycobacterium*; MIRU-VNTR, mycobacterial interspersed repetitive unit variable number of tandem repeats; WGS, whole-genome sequencing ^a^ MIRU-type and whole genome sequencing-derived phylogenetic tree and single nucleotide polymorphism-based distance matrix for all putative contamination isolates. Clustered isolates are highlighted in orange for both typing system results ^b^ H37Rv is the *M. tuberculosis* Lineage 4 reference strain

The ability and relative resolution of both molecular typing systems to further support, or refute, laboratory cross-contamination were assessed. Laboratory procedures at higher risk of cross-contamination were also identified. Cross-contamination was initially suspected when patients without clinical symptoms and epidemiological profiles compatible with TB had positive samples (patients 1, 2 and 3). Despite testing positive by culture, these patients’ primary samples were all negative on smear microscopy and, when available, their follow-up samples were all culture negative, reducing the likelihood of true positive culture results. On the contrary, the results obtained for patients 4 and 5 were considered as potential true positives since these patients had previously lived or transited in TB endemic settings and presented with TB-compatible clinical symptoms. Moreover, patient 5’s primary samples were also smear positive (n=3) and NAAT positive (n=2, one sample not tested), and all of their follow-up cultures were also positive, increasing the likelihood of a true positive result. Clinical characteristics and sample trajectories of all patients are reported in **Table 1**. Whole-genome sequence phylogenetic analysis and MIRU-VNTR typing results are presented in **Figure 1**.

**Table 1:** Patients’ clinical presentation and laboratory testing results for *Mycobacterium tuberculosis* pseudo-outbreak

Patient	Clinical presentation	Sample	Sample laboratory trajectory (day)					Complementary laboratory results		
			Sampling	Culture media inoculation	Positive growth	Positive culture media	Reference laboratory send out	PCR	AFB staining	Follow-up cultures and PCR
1	Organ donor	Pre-transplant mycobacterial culture	0	1^a^	20	21^a^	24^a^	Not done	Negative	Negative
2	Intravenous drug user with skin abscess	Superficial wound culture	−1	1^a^	43	32	52	Not done	Negative	Negative
3	Chronic pneumonia	Bronchial aspiration	−3	1^a^	17	18	24^a^	Positive	Negative	Negative
4	Hypermetabolic lung nodules on PET CT	Bronchoalveolar lavage	−12	−12	16	21^a^	24^a^	Negative	Negative	Negative
5	Apical lung consolidation and cavitation	Induced expectorations (a, b, c)	−3	0^a^	6 (a, b) & 7 (c)	8 (a, b) & 9 (c)	10	Positive (b, c)	Positive (a, b, c)	Positive
6	N/A	Costal bone sample	2013	2013	2013	2013	2013	Negative	Negative	N/A

Abbreviations: AFB, acid fast bacilli; N/A, not applicable; PCR, polymerase chain reaction; PET CT, positron emission tomography computerized tomodensitometry

^a^ Possible cross-contamination events due to overlapping laboratory procedures. Samples from patients 1, 2, 3 and 5 were received, processed and inoculated on culture media in the same period. Positive mycobacteria growth indicator tube (MGIT) culture media from patients 1 and 4 were opened, aliquoted and smeared on the same day. Samples from patients 1, 3 and 4 were aliquoted and referred to the reference *Laboratoire de santé publique du Québec* (LSPQ) on the same day. Patient 6 was included in the investigation at a later stage since his bacterial isolate was sharing the same mycobacterial interspersed repetitive unit (MIRU)-type as the cluster and this patient’s sample had previously been processed in our laboratory

Isolates from patients 1, 2, 3 and 5 had identical MIRU-type and WGS confirmed those isolates to be genetically identical (0 single nucleotide polymorphism [SNP] difference). The isolate from patient 4 had a different MIRU-type and WGS confirmed a significant genetic distance from the other isolates. We retrospectively identified only one bacterial isolate from our laboratory that shared the same MIRU-type as the cluster (patient 6). This patient’s isolate was cultured in 2013 from a costal bone sample. Despite sharing a MIRU-type, WGS showed this isolate to have a 13- to 16-SNP distance with the other clustered isolates. Sequencing quality metrics are available in the supplementary materials. Interestingly, this patient had immigrated from the same TB endemic country as patient 5. Confronting epidemiological, clinical and genomic data allowed confirmation that cross-contamination had occurred. Given the convincing epidemiological context, clinical manifestations, stronger smear positivity and positive follow-up cultures, samples from patient 5 were considered to be true positive samples and to represent the index sample or the source of contamination. With identical genotyping but absence of, or incompatible symptomatology and subsequent negative TB test results, patients 1, 2 and 3 were considered to have false positive culture due to cross-contamination. Patient 4, however, was considered TB positive, given the distinct MIRU-type and WGS results.

Patient 2 was never started on TB treatment, given the rapid clinical improvement with anti-staphylococcal therapy. Patient 3 was initially started on TB treatment, which was discontinued after four weeks, as soon as the hypothesis of laboratory contamination was raised. As stated above, three patients had received organs from patient 1 (lungs, heart and kidney). They were all initially presumed as having active TB when the donor’s sample became culture positive. Given the high risk of unfavourable outcomes in the context of immunosuppression, they were then started on therapy for active TB and had serial follow-ups with infectious disease clinicians. None of them developed TB symptoms. Three months later, when complemented by molecular typing, the results of this investigation were deemed sufficiently convincing by the clinicians to discontinue TB therapy among these organ transplant recipients.

## Discussion

As previously reported, cross-contamination of *M. tuberculosis* cultures is not an uncommon event ((5,6)). A comprehensive and modern approach to investigate *M. tuberculosis* laboratory cross-contamination is presented, including molecular typing, which proved to be the cornerstone in confirming sample contamination directionality. Within the cluster of positive samples examined, WGS showed higher typing resolution than MIRU-VNTR, as was previously reported ((15,16)). Our investigation led to discontinuation of potentially toxic TB treatments for multiple patients, including immunocompromised transplant recipients for which the implications of a positive TB diagnosis are even more significant ((16,17)).

*Mycobacterium tuberculosis* culture cross-contamination may result from its intrinsic ability to create aerosols that survive for extended periods in air and harsh environments, as well as some specific laboratory techniques. In an article published in 2019, the three leading causes of cross-contamination in *M. tuberculosis* culture were human technical errors by laboratory technicians, contamination of reagents and aerosol production ((5)). An important step in the culture process is decontamination. During this step, bactericidal buffer agents are added to the clinical sample to kill bacterial and fungal flora ((18)). This technique is specifically prone to cross-contamination. It is usually carried out on specimen batches, as it implies multiple timed steps. Contamination of one of the reagents, often the neutralizing buffer, leads to the inoculation of *M. tuberculosis* in the subsequently processed samples ((19)). In our laboratory, 14 samples can be decontaminated at a time and we established that contamination of the buffer vial was the most probable vector. This vial could be used over multiple days, leading to possible contamination between samples being decontaminated on separate days, as suspected in our current investigation report. The two other steps where contamination could have occurred are at the opening of positive MGIT media for acid-fast smear growth confirmation and aliquoting prior to sending the sample out to the reference laboratory. Aerosols may be generated during these procedures, since bottles are being reopened near one another. Different methods can be used to reduce the risk of contamination, such as reducing the number of processed samples per batch, disinfecting the common buffer vial between each sample, employing single-use materials and dispensed reagents, using centrifuge caps to limit aerosol production, regular staff training and integrating a negative control in each batch of samples tested ((12)).

Other reports of *M. tuberculosis* cross-contamination and their investigation are available in literature ((6–8,10)). The systematic review published in 2019 identified 31 articles describing *M. tuberculosis* cross-contamination events and described the different genotyping methods used. Most of these studies used conventional molecular typing methods, such as IS6110-RFLP, MIRU-VNTR, spoligotyping and direct repeat (DR)-RFLP typing. However, no article in the literature had previously used the nanopore NGS platform for this specific application. In our sample cluster, the latter showed higher typing resolution as one patient with TB reported in 2013 had the same MIRU-type, but clustered with less than 20 SNPs. This patient had immigrated from the same sub-Saharan African country as the pseudo-outbreak index patient, but no epidemiological link could be established between both patients.

## Limitations

As in many laboratory contamination events, the success of our investigation relied on initial clinical suspicion that false positive results had been reported. Our investigation then relied on modern genotyping methods, which are becoming more widely available, but still aren’t easily accessible everywhere. This is even more of a concern in low-income countries, where *M. tuberculosis* is more prevalent and where sequencing technologies are underutilized ((20)). Our laboratory serves a population where *M. tuberculosis* incidence is low. In high burden settings, mixed infection and higher active transmission could make it more difficult to clinically identify potential contamination events ((21,22)).

## Conclusion

This investigation highlights the ongoing risk of *M. tuberculosis* cross-contamination in mycobacteria laboratories, including in high-income settings. Combining epidemiological, clinical and molecular data can help resolve contamination events and optimize patient care. *Mycobacterium tuberculosis* WGS shows higher typing resolution than MIRU-VNTR, but typing results from both methods can improve our understanding of contamination directionality between clustered positive samples. Different approaches and methods should be taken to reduce the risk of contamination.
